# Supplementary material for: Oncogenic mutation or overexpression of oncogenic KRAS or BRAF is not sufficient to confer oncogene addiction
Source: PLoS One. 2021 Apr 1;16(4):e0249388. doi: 10.1371/journal.pone.0249388 (PMC8016361; doi:10.1371/journal.pone.0249388)

Figure 2F_raw

800 nm emission

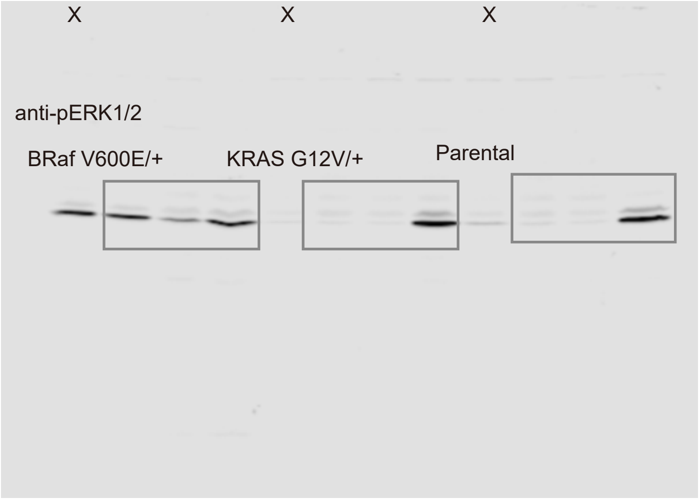

680 nm emission

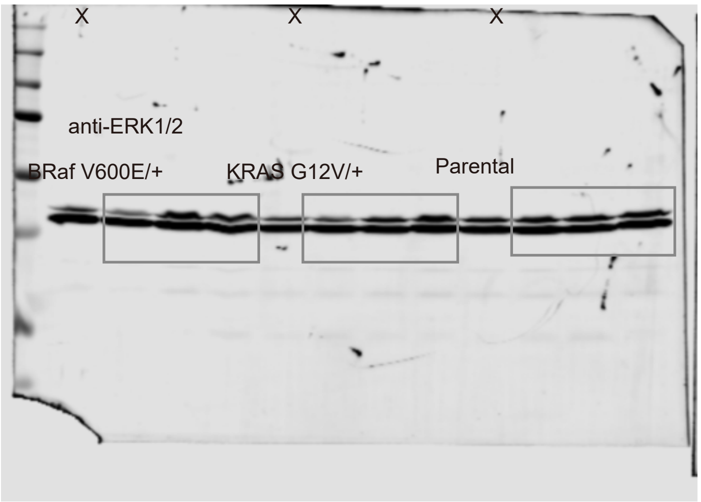

Figure 4F_raw

800 nm emission

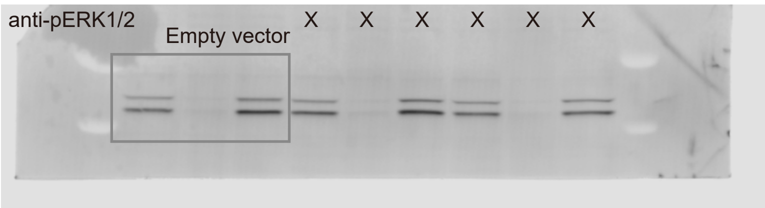

680 nm emission

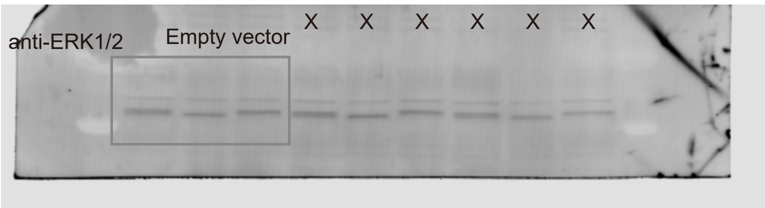

800 nm emission

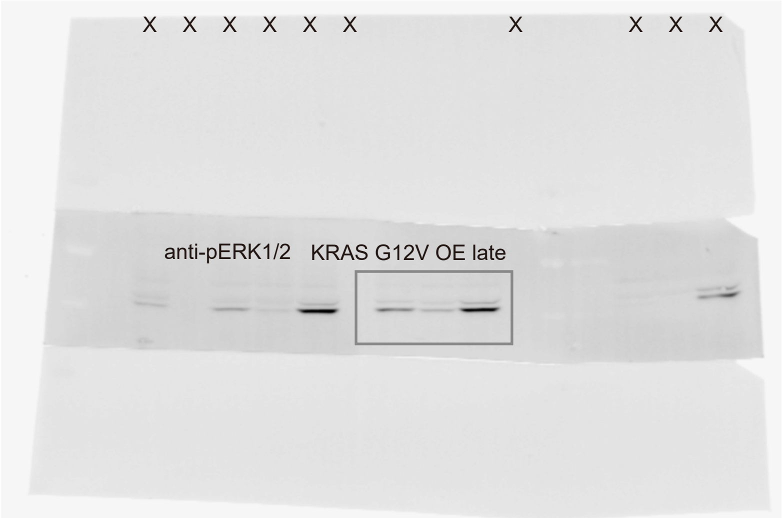

680 nm emission

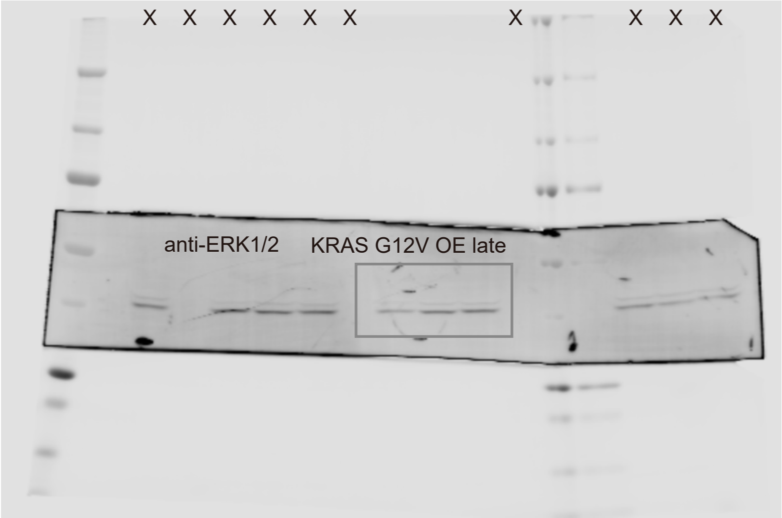

800 nm emission

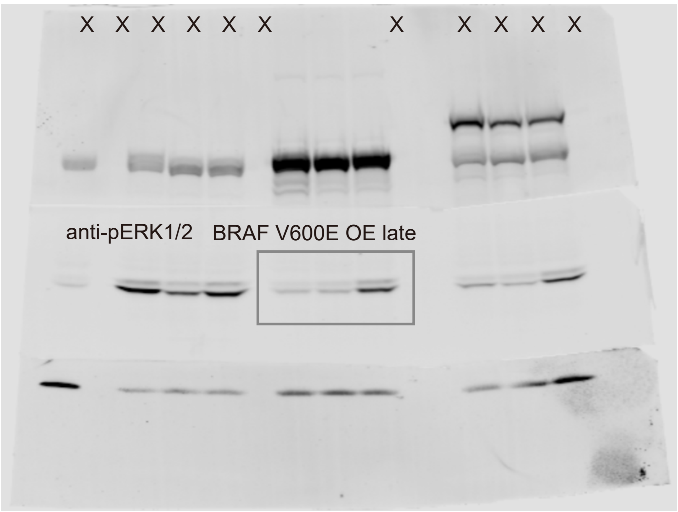

680 nm emission

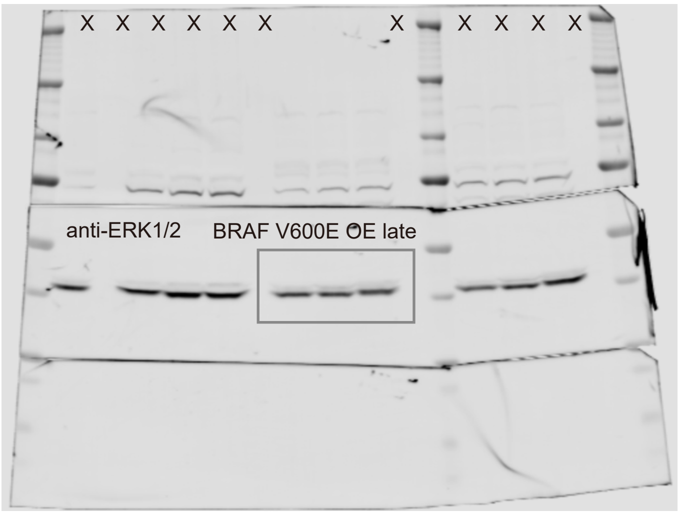

Figure S1C_raw

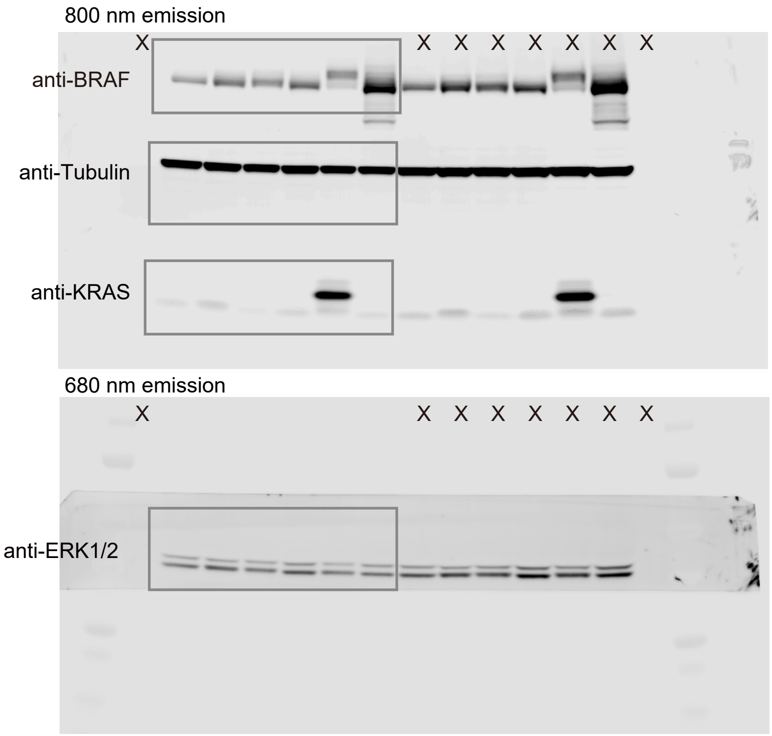

Figure S2A_raw

800 nm emission

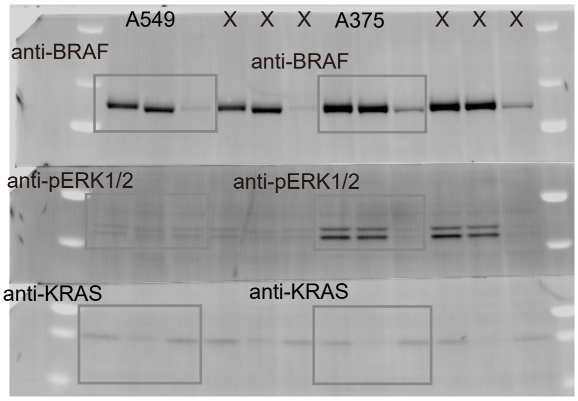

800 nm emission

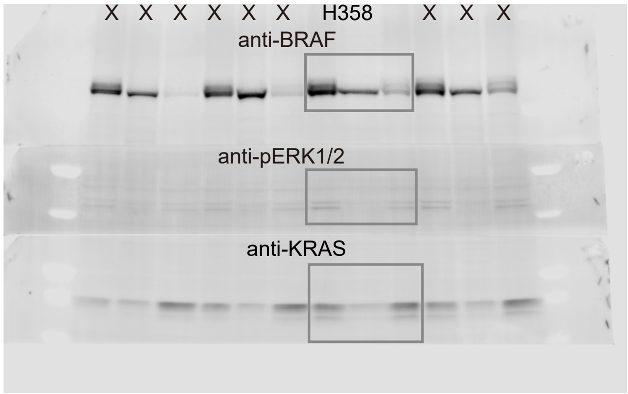

680 nm emission

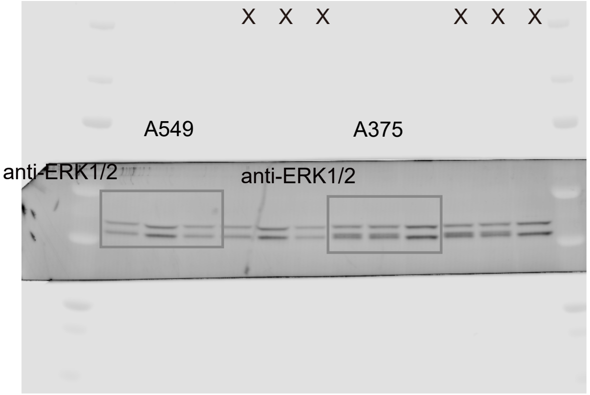

680 nm emission

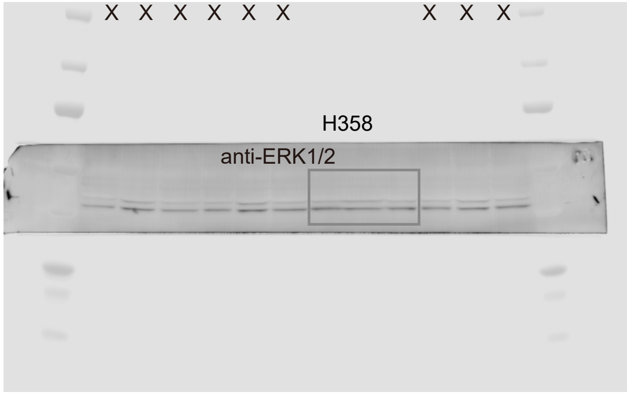

Figure S2C_raw

800 nm emission

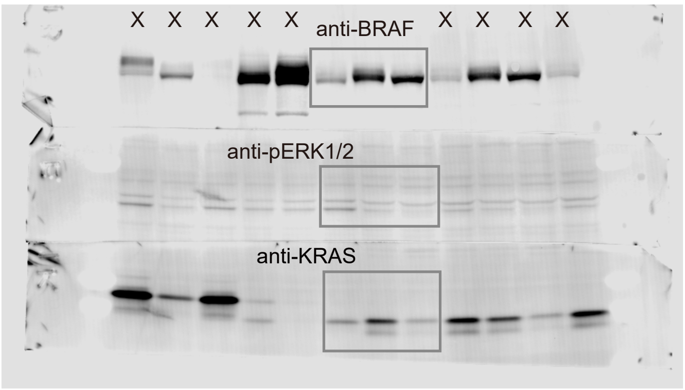

680 nm emission

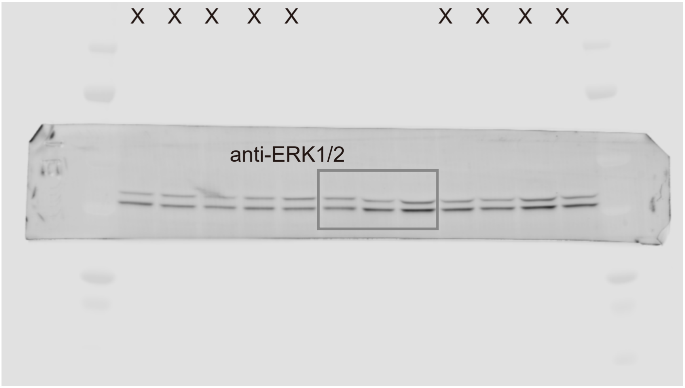

Figure S3A_raw

800 nm emission

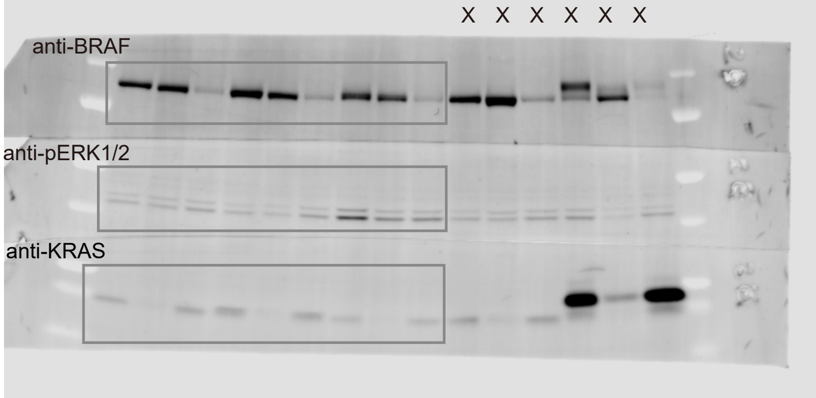

680 nm emission

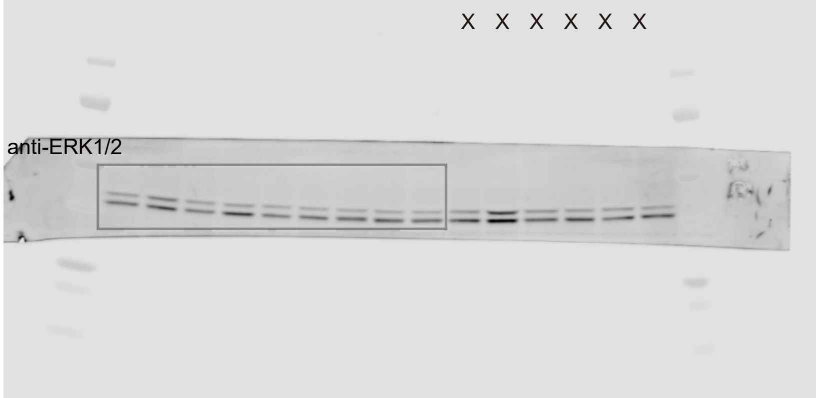

Figure S3C_raw

800 nm emission

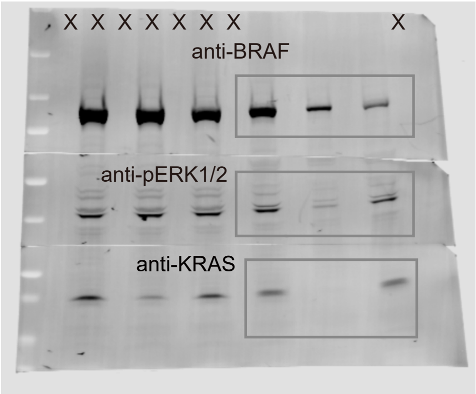

800 nm emission

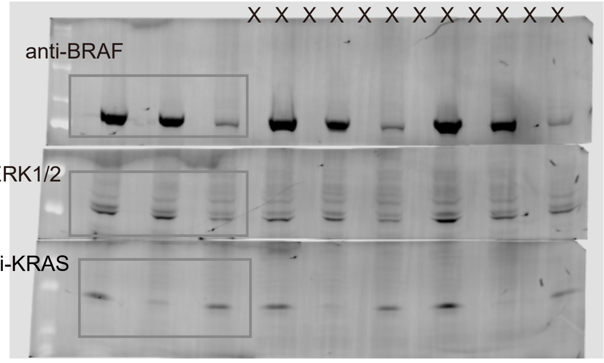

680 nm emission

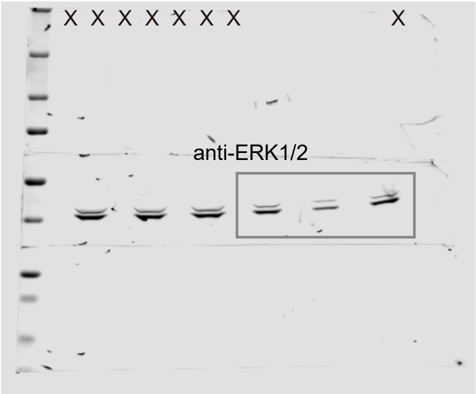

680 nm emission

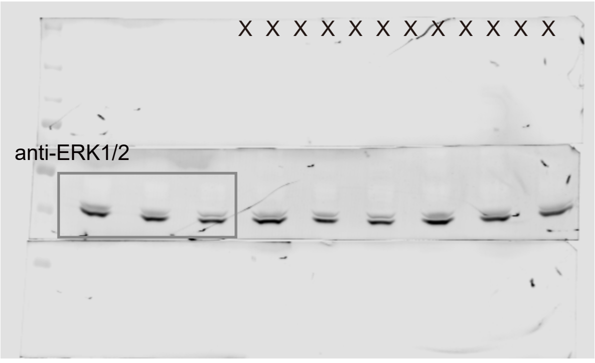

Figure S4A_raw

800 nm emission

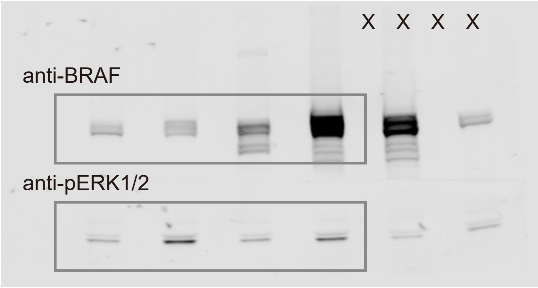

680 nm emission

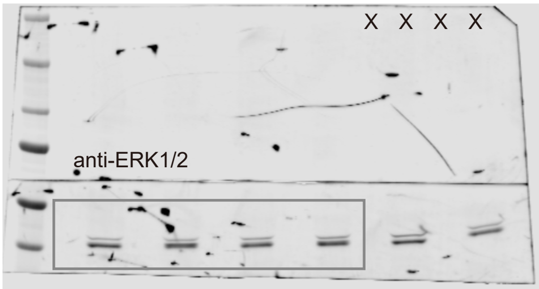

Figure S5A_raw

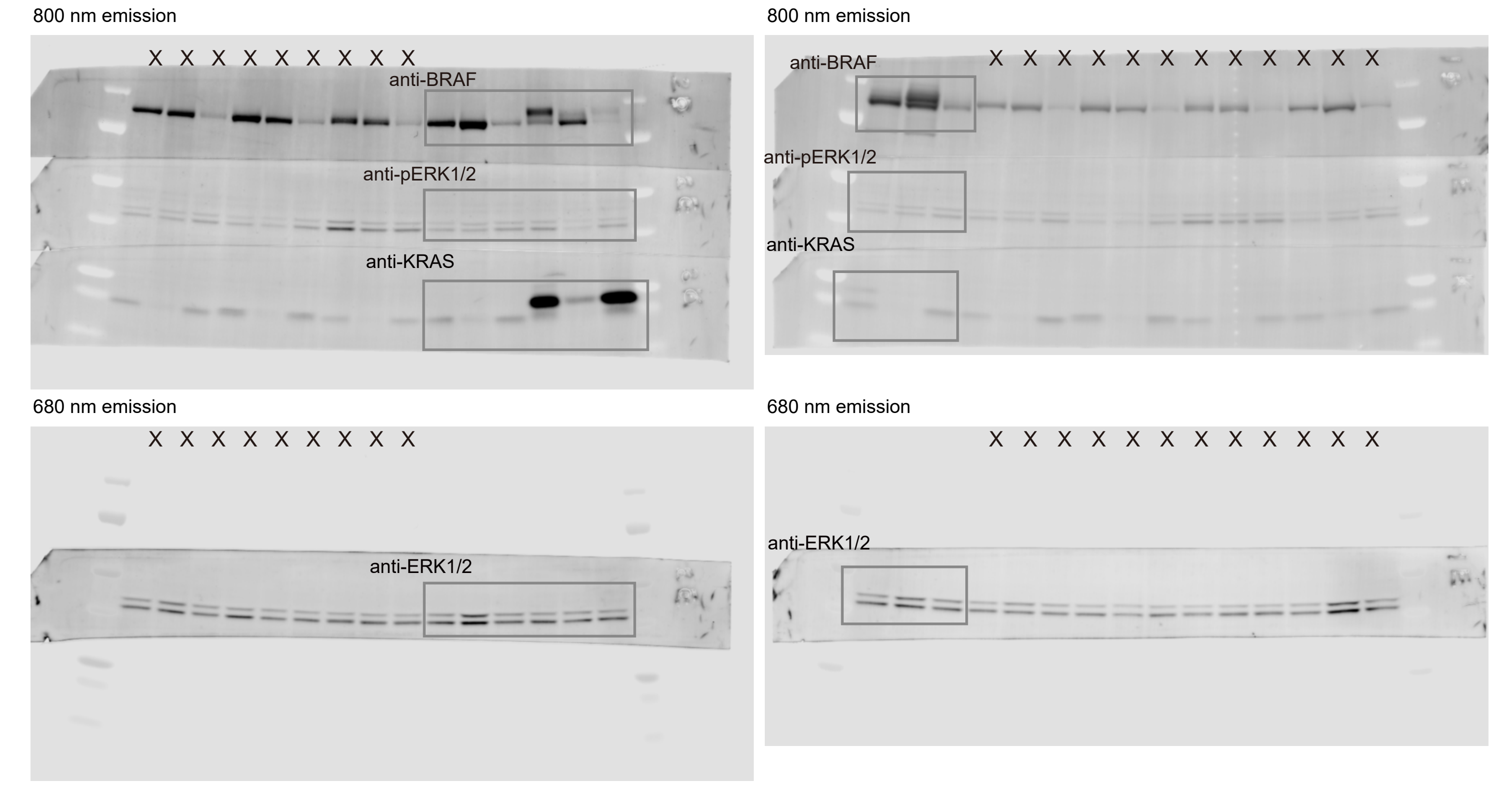

Figure S5C_raw

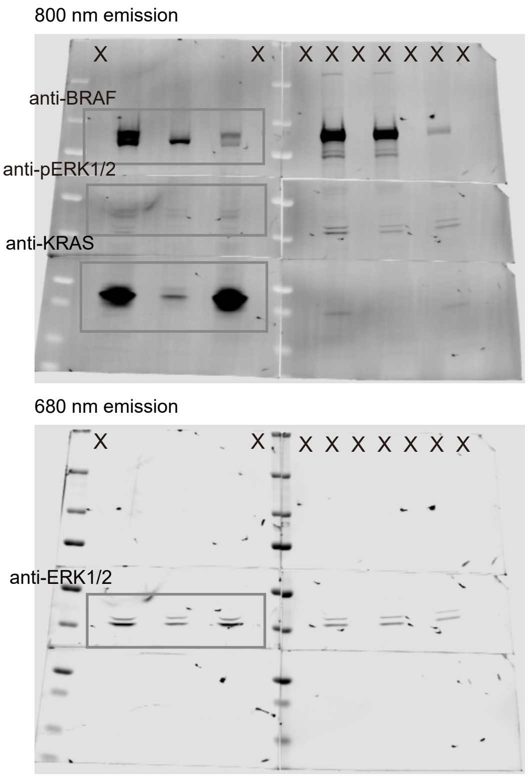

Supplement: S1 Raw images — (PDF) [file pone.0249388.s006.pdf]
